# Effect of Anti-COVID-19 Mouthwashes on Shear Bond Strength of Resin-Matrix Ceramics Repaired with Resin Composite Using Universal Adhesive: An In Vitro Study

**DOI:** 10.3390/jfb14030158

**Published:** 2023-03-16

**Authors:** Wichuda Limsiriwong, Awiruth Klaisiri, Nantawan Krajangta

**Affiliations:** 1 Division of Restorative Dentistry, Faculty of Dentistry, Thammasat University, Pathum Thani 12120, Thailand; 2 Thammasat University Research Unit in Restorative and Esthetic Dentistry, Thammasat University, Pathum Thani 12120, Thailand

**Keywords:** anti-COVID-19, hybrid material, mouthwash, repaired, shear bond strength

## Abstract

Using anti-COVID-19 mouthwashes has become necessary to reduce acute respiratory syndrome coronavirus 2 (SARS-CoV-2) transmissions. Resin-matrix ceramic (RMCs) materials that are exposed to mouthwashes may affect the bonding of repaired materials. This research was performed to assess the effects of anti-COVID-19 mouthwashes on the shear bond strengths (SBS) of RMCs repaired with resin composites. A total of 189 rectangular specimens of two different RMCs (Vita Enamic (VE) and Shofu Block HC (ShB)) were thermocycled and randomly divided into nine subgroups according to different mouthwashes (distilled water (DW), 0.2% povidone–iodine (PVP-I), and 1.5% hydrogen peroxide (HP)) and surface treatment protocols (no surface treatment, hydrofluoric acid etching (HF), and sandblasting (SB)). A repair protocol for RMCs was performed (using universal adhesives and resin composites), and the specimens were assessed using an SBS test. The failure mode was examined using a stereomicroscope. The SBS data were evaluated using a three-way ANOVA and a Tukey post hoc test. The SBS were significantly affected by the RMCs, mouthwashes, and surface treatment protocols. Both surface treatment protocols (HF and SB) for both RMCs, whether immersed in anti-COVID-19 mouthwash or not, improved the SBS. For the VE immersed in HP and PVP-I, the HF surface treatment had the highest SBS. For the ShB immersed in HP and PVP-I, the SB surface treatment had the highest SBS.

## 1. Introduction

Coronavirus disease 2019 (COVID-19) is considered to be caused by the severe acute respiratory syndrome coronavirus 2 (SARS-CoV-2) and is transmissible between humans via multiple pathways. The main transmission pathways are droplet transmission and contact transmission. In a closed setting exposed to a high concentration of aerosol, the possibility of aerosol transmission exists [1,2].

As routine dental procedures create aerosols, a preoperative antiseptic is recommended [3,4,5]. Due to the presence of SARS-CoV-2 in nasopharyngeal secretions and saliva, the oral cavity can be an important source of infection. This has been supported by findings on the detection of viral loads in the saliva samples of COVID-19 patients. A total of 91.7 percent of the saliva samples were found to contain the virus, and the infective copies/mL can be as high as 1.2 × 10^8^ [2]. The salivary glands and the throat are other sites for SARS-CoV-2 infection [6]. Accordingly, decreasing the viral load in the oral cavity should reduce the incidence of COVID-19 transmissions, and mouthwash is recommended for this purpose.

The National Health Commission of the People’s Republic of China has published in the fifth issue of the Guideline for the Diagnosis and Treatment of Novel Coronavirus Pneumonia that chlorhexidine (CHX), a common antiseptic in mouthwash, cannot effectively inactivate the virus [4]. Since the virus is susceptible to oxidation, mouthwashes containing oxidative agents, such as povidone–iodine or hydrogen peroxide, appear to be anti-virucidal [4].

Computer-aided design/computer-aided manufacturing (CAD/CAM) has been developed to advance the fabrication of ceramic restorations and has become a popular method in dentistry. CAD/CAM indirect restorative materials can be classified into ceramics and composites [7]. Ceramics are recommended in dental practice mostly for restoration, although they possess low fracture toughness and high brittleness [8]. To improve these physical defects, resin-matrix ceramic (RMCs) materials were developed to (a) produce a material that more closely resembles dentin’s modulus of elasticity than traditional ceramics, (b) be simpler to mill and adjust than polycrystalline ceramics and glass-matrix ceramics, and (c) promote repair or adjustment with resin composites. These factors justify the enhanced physical and mechanical properties of ceramic materials containing a resin matrix. This makes them popular alternative materials in restorative dentistry [9].

Regardless of the materials, the major disadvantage of CAD/CAM ceramic restoration materials is fractures [10,11]. To repair only a minor fracture, a composite resin can alternatively be applied, which provides less complexity and lower costs [12]. However, replacement restoration is not recommended as it is time-consuming, invasive, and causes discomfort for the patients [13].

According to Silva and Botta [14], hydrogen peroxide can negatively affect bond strength by creating high porosity levels, reducing mineral contents and prisms in the enamel, and creating more fragmented resin tags. In addition, Demir and Malkoc [15] stated that chlorhexidine and povidone–iodine have no significant impact on the bond ability of orthodontic composites to the enamel surface and also increase the shear bond strength (SBS) when treating enamel. Numerous studies [16,17,18,19,20,21] have investigated the effects of alcohol-containing, alcohol-free, and chlorhexidine-containing mouthwashes on the surface roughness and SBS of resin-based composite restorations. However, studies on the influence of anti-COVID-19 mouthwashes on the shear bond strengths of aged RMCs repaired with resin composites are limited and unexplained. Thus, the study objective was to evaluate the effects of two different resin-matrix ceramics, two different types of anti-COVID-19 mouthwashes, and two different surface treatment protocols on the shear bond strengths of RMCs repaired with resin composites when using a universal adhesive. The first, second, and third hypotheses were that the SBS of the RMCs and composites were affected by the RMCs types, anti-COVID-19 mouthwash types, and surface treatment protocols, respectively.

## 2. Materials and Methods 

### 2.1. Specimen Preparation

**Figure 1:**
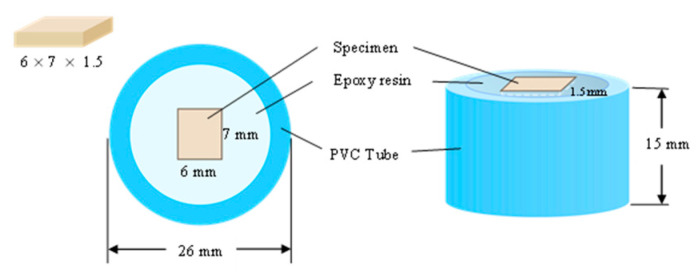
RMCs specimen preparation.

The 189 rectangular specimens per material were produced from VITA Enamic (VITA Zahnfabrik, Bad Sackingen, Germany) and Shofu Block HC with a thickness of 1.5 mm (Shofu Inc., Kyoto, Japan). The specimens were encased in a PVC tube filled with an epoxy resin (Figure 1). All samples were polished using an automatic polishing device with a 1200-grit silicon carbide paper (3M Wetordry abrasive paper; 3M, St. Paul, MN, USA), a polishing disk running at 300 rounds/minute, and a holder plate with a co-rotation of 150 rounds/minute for 60 s under running water (Tegramin-25; Struers. Inc., Cleveland, OH, USA) [22]. The specimens were then cleaned ultrasonically (GA008G-60W; Thai C.L.H., Bangkok, Thailand) for 5 min with distilled water. The samples were transmitted to a thermal cycler for 5000 thermal cycles between 5 °C and 55 °C with a dwell time of 30 s and a transfer time of 5 s.

All samples were randomly grouped into three groups (n = 63 each) based on their immersion solutions as follows: Group 1, distilled water (control); Group 2, 0.2% povidone–iodine (PVP-I); Group 3, 1.5% hydrogen peroxide (Siribuncha^®^, Siribuncha corporation, Bangkok, Thailand).

### 2.2. Storing in Anti-COVID-19 Mouthwash

Two anti-COVID-19 mouthwashes, 0.2% povidone–iodine (PVP-I) and 1.5% hydrogen peroxide (Siribuncha^®^, Siribuncha corporation, Bangkok, Thailand) [4], and distilled water (control group) were used in this study. The 0.2% PVP-I was prepared using a ratio of 30:1 of distilled water and 7% betadine gargle (BETADINE^®^ Mouthwash/Gargle, Thai Meiji Pharmaceutical, Bangkok, Thailand). The 1.5% hydrogen peroxide was prepared using a ratio of 1:1 of distilled water and 3% hydrogen peroxide (Siribuncha^®^, Siribuncha corporation, Bangkok, Thailand) [4]. The distilled water was used in the form in which it was delivered.

The obtained material specimens were randomly separated into three subgroups (N = 63) and submerged in 400 mL of the anti-COVID-19 mouthwashes (0.2% PVP-I or 1.5% hydrogen peroxide) or distilled water (control groups) for 30 s.

### 2.3. Surface Treatment and Repair Procedure

The sixty-three specimens of each RMCs and immersion solution were divided into three subgroups based on the surface treatment protocols. The samples were then subjected to one of the subsequent surface modification protocols (N = 21) as follows: 

Control group: no surface treatment.

Hydrofluoric acid (HF) group: etching with 9% HF (Ultradent Porcelain Etch; Ultradent Products, South Jordan, UT, USA) for 60 s. The HF was first washed with distilled water and then gently aerosolized.

Sandblasting (SB) group: airborne-particle abrasion that uses a sandblasting machine (A10723 Base 3; Dental Vision Co. Ltd., Bangkok, Thailand) that is 10 mm parallel to the specimen surface at a bar pressure of 2 and aluminum oxide with a size of 50 microns for 15 s. The specimens were immersed in an ultrasonic bath with distilled water for 5 min before being gently aerosolized.

A universal adhesive (Scotchbond Universal Plus; 3M ESPE, St. Paul, MN, USA) was applied to the specimen’s surface for 20 s using a microbrush. After that, a new microbrush was used to eliminate the excess adhesive inside the mold, and the adhesive was air-dried for approximately 10 s to evaporate the solvent [23] from the surface of the specimen, after which it was air-dried until there was no fluid flow and the surface was glossy and smooth. Finally, the adhesive was exposed to light for 20 s using a light-emitting diode (LED) curing device (Elipar S10; 3M ESPE, St. Paul, MN, USA).

**Figure 2:**
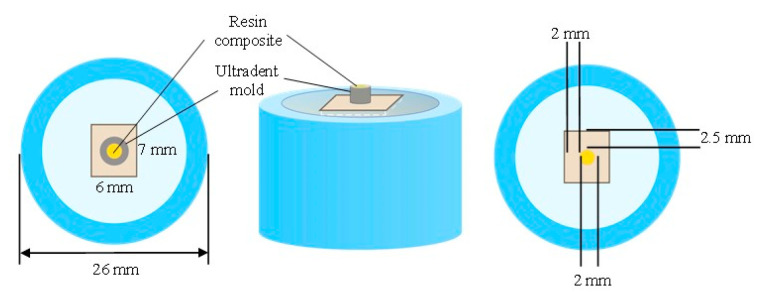
A resin composite bonded to the RMCs specimen.

After the universal adhesive application, a resin composite (Filtek Z350 XT; 3M ESPE, St. Paul, MN, USA) was bulk filled onto the treated hybrid material surfaces using a plastic mold (Ultradent Product Inc., South Jordan, UT, USA) with a diameter of 2.0 mm and a height of 2.0 mm (Figure 2). It was lightly cured for 40 s using an LED light-curing device. The light-curing unit was perpendicular and as close to the specimen as possible. After curing, the plastic mold was removed, and then an additional 40 s of light curing was performed at the adhesive interface. Finally, the bonded samples were placed in distilled water at 37 °C for 1 day.

**Figure 3:**
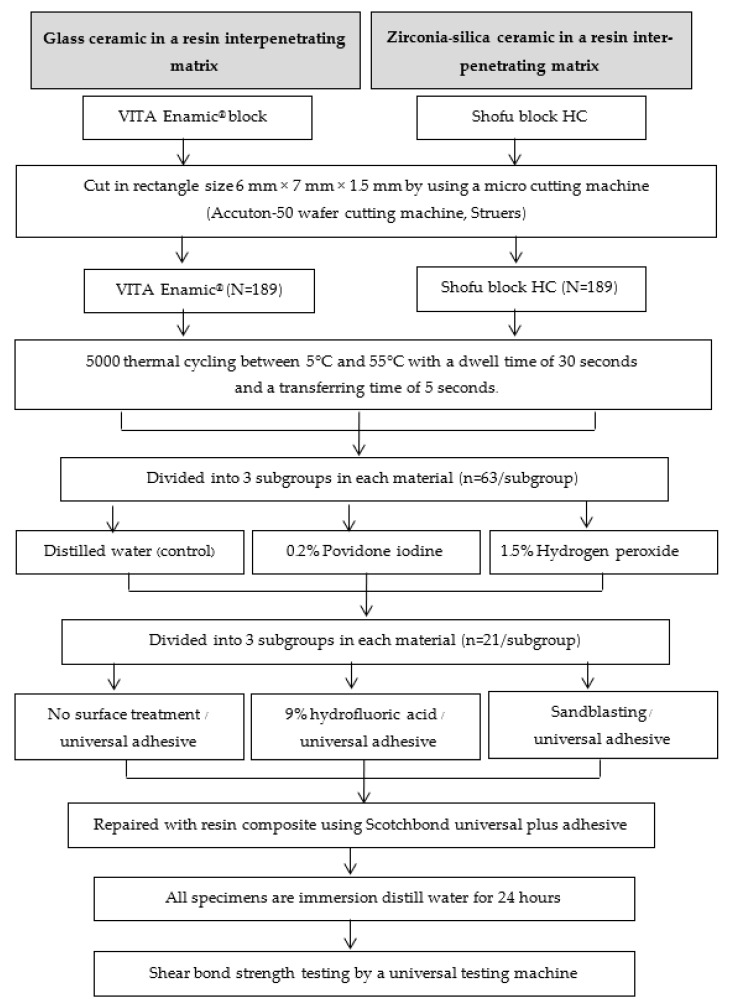
Experimental design.

The chemical material compositions used in this research are displayed in Table 1, and the experiment design is presented in Figure 3.

### 2.4. SBS Test 

**Figure 4:**
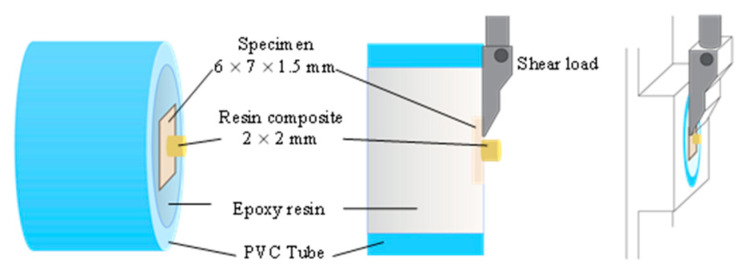
SBS test setup.

For the SBS tests, the bonded samples were fixed to a universal testing apparatus (AGS-X 500N; Shimadzu Corporation, Kyoto, Japan). The bonded samples were secured in the testing apparatus, and the shearing blade was positioned 1 mm above and parallel to the junction between the resin-matrix hybrid material and the resin composite (Figure 4). The shear load was applied at a 0.5 mm/min crosshead speed until failure. The SBS values (MPa) were computed by dividing the failure load by the interface of the bonded surface area [26,27]. 

### 2.5. Mode of Failure Analysis

After determining the SBS, the de-bonded resin-matrix hybrid material surfaces were analyzed using a stereomicroscope at 35× to determine the mode of failure, which consists of the following three types [24]:

(a) Adhesive failure: over 75% of the failure occurs at the interface between the resin-matrix hybrid material and the universal adhesive.
(b) Cohesive failure: over 75% of the failure occurs within the universal adhesive or composite.
(c) Mixed failure: 25–75% of the failure occurs as a combination of cohesive and adhesive failure.

### 2.6. Statistical Analysis

Using the SPSS 26.0 software for Mac (SPSS Inc., Chicago, IL, USA) and a confidence level of 95%, the results for all groups were determined. First, the normality of the distribution was investigated using the Kolmogorov–Smirnov (KS) test, and the homogeneity of the variance was determined using the Levene test. The SBS values were then evaluated using a three-way ANOVA, followed by a Tukey’s post hoc test to assess multiple comparisons.

## 3. Results

The three-way ANOVA (Table 2) results revealed that the material type, mouthwash, and surface treatment protocol had significant effects on the SBS values, with *p* < 0.01. There was a significant interaction for material * mouthwash (*p* < 0.01), mouthwash * surface treatment (*p* < 0.01), and material * surface treatment (*p* < 0.05). Additionally, there was a significant three-factor interaction for material * mouthwash * surface treatment (*p* < 0.01).

**Figure 5:**
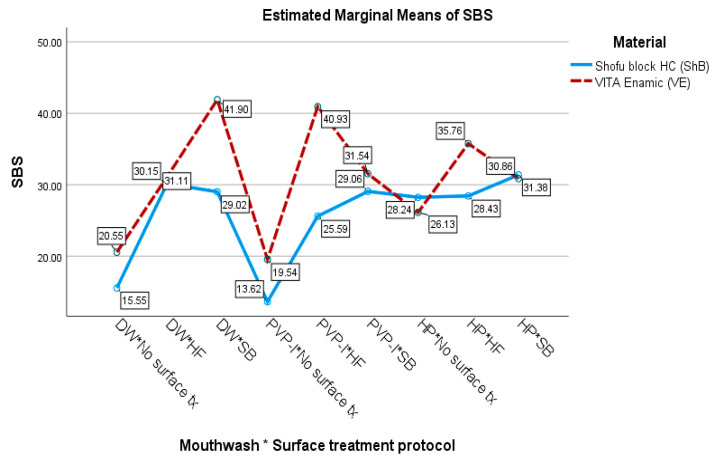
Mean SBS for each mouthwash * surface treatment protocol (two factor interaction) according to material type.

**Figure 6:**
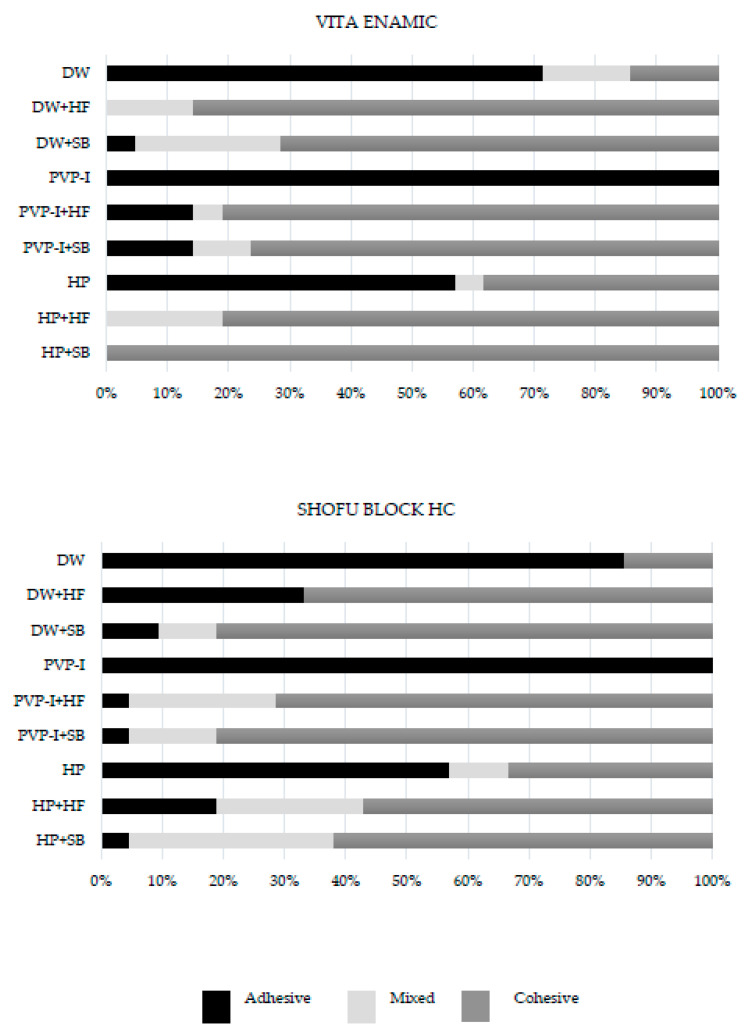
Distribution of failure mode for each group (%) subjected to SBS.

In the present investigation, the means and standard deviations of the SBS are shown in Table 3. For the VE, whether immersed in DW, HP, or PVP-I, the SB was the highest with the HF surface treatment, while for the ShB that was immersed in HP and PVP-I, the SB surface treatment had the highest SBS (Figure 5). The distribution of the failure mode for each group is presented in Table 3 and Figure 6.

The dominant failure mode was adhesive for both VE and ShB when there was no surface treatment. On the other hand, for both VE and ShB, when their surfaces were treated with sandblasting and HF, they showed predominantly cohesive failure in the composites.

## 4. Discussion

At present, the RMCs are frequently used for several reasons. They are more flexible and less abrasive on a patient’s teeth [28]. They are not brittle and are easy to manipulate for intraoral repairs [29]. Defective RMCs can be repaired with resin composites in the dental office, which is less costly and more conservative, with less loss of dental structure and less irritation to pulpal tissues compared to a replacement with new materials [13,30]. This study assessed the SBS of two types of aged RMCs after immersion in two types of anti-COVID-19 mouthwashes, followed by two procedures of surface treatments and then repairs with resin composites using universal adhesives. An SBS test was used to investigate the bond ability, which is a simple and common method used for the assessment of bond strength repairs [31,32,33,34,35,36]. Based on the difference in composition, the following two types of RMCs were used in this study: VE, a polymer-infiltrated ceramic network (PICN), and ShB, a dispersed filler resin block (resin nanoceramic) [37]. A researcher accelerated the aging of the RMCs by 5000 cycles of thermocycling, which is equivalent to half a year of clinical use [38]. The effects of the 0.2% PVP-I and 1.5% HP anti-COVID-19 mouthwashes on the SBS of the repaired RMCs were assessed in this study because they were recommended by the American Dental Association (ADA) to be used as a prior dental procedure during the COVID-19 pandemic [39]. Additionally, the effects of the different surface treatments for RM, including chemical etching with HF and sandblasting with aluminum oxide, were also evaluated in this study. The same adhesive (Scotchbond Universal Plus) and resin composite (Filtek Z350 XT) were used in the repair process to control the confounding factor and repair the SBS. 

Regarding the results of this study, the SBS of the repaired RMCs with the resin composites were affected by the RMCs types, anti-COVID-19 mouthwash types, and surface treatment protocols. Therefore, all three hypotheses were rejected. The acceptable repair bond strength has no standard requirements. According to previous reports, a repair bond strength value of at least 20 MPa is clinically optimum [40,41]. This study found that the repair SBS values of both RMCs types, whether immersed in anti-COVID-19 mouthwash or not, and when surface treated with both SB and HF, ranged from 29.02–41.90 MPa and were clinically acceptable. Similar results for the repair SBS values were obtained for the VE and ShB without a surface treatment and when immersed in HP (26.13 and 28.24 MPa, respectively), and the values were clinically acceptable. On the other hand, the repair SBS values of both RMCs types when immersed in DW and PVP-I without surface treatment were insufficient to be clinically acceptable.

Among the different RMCs, the results showed that the SBS of the repaired VE was higher than that of ShB, which is in agreement with many previous studies [42,43,44]. The explanation for the higher SBS of the repaired VE might be related to the effect of the RMCs composition. For VE, the inorganic phase was a glass ceramic in a resin-interpenetrating matrix, while for ShB the inorganic phase was a zirconia-glass ceramic in a resin-interpenetrating matrix. The glass ceramic in VE had better bondability to the composite when compared with the zirconia-glass ceramic in ShB [45]. 

During the COVID-19 pandemic, the use of anti-COVID-19 mouthwash before dental procedures was recommended. Regarding the three-way ANOVA results in the RMCs repair approach, the surfaces of the aged RMCs, which were exposed to the mouthwash, influenced the SBS of the RMCs that were repaired with the resin composites, regardless of the RMCs type or surface treatment procedure. The aged RMCs that were immersed in 0.2% PVP-I had the lowest (13.62–40.93 MPa) repaired SBS, followed by DW (15.55–41.90 MPa), while the 1.5% HP showed the highest (26.13–35.76 MPa) repaired SBS. This study revealed the difference between the PVP-I and HP based on the SBS of the repaired RMCs. Tanthanuch et al. reported that PVP-I and HP affect the roughness and color change of the composite resins due to their ability to diffuse through the polymer network [46]. Therefore, the PVP-I and HP mouthwashes may be able to diffuse through the polymer matrix of the RMCs and then affect the SBS. The reduction in the SBS of the repaired RMCs after immersion in PVP-I might result from the presence of glycerol in the composition of PVP-I, which was incompletely removed from the RMCs surface before the repair procedure. Glycerol is highly viscous and soluble in water only after extensive time [47]. The residual glycerol might interfere with the adhesion between the aged RMCs and the composite. In this study, we found the greatest SBS value in the repaired RMCs after immersion in HP. Even HP can diffuse through the RMCs and dissociate the free radical peroxides, which can disrupt polymerization and reduce the SBS. However, the HP mouthwash concentration was relatively low at 1.5%. Tanthanuch et al. found that the acidity of the 1.5% HP could promote greater roughness in the resin composite surface, which may result in increased mechanical retention to the RMCs surface and promote greater repair SBS as a result of this study [46].

The results of this study showed that the surface treatment procedures, both HF and SB, increased the SBS before the repair of the RMCs when compared to those without a surface treatment group. The creation of a bond between the freshly added resin composite and the aged RMCs is more difficult because the RMCs has a CAD/CAM restoration industrial polymerization with a high degree of conversion and insufficient free radicals. Therefore, a surface treatment of the aged RMCs before the resin composite repair was necessary to improve the SBS [48]. The result revealed not only the surface treatment effects on the repaired SBS of the RMCs but also the different effects of the surface treatment protocols (HF and SB) on the SBS according to the type of RMCs. In the repaired VE, the SBS was more enhanced by the surface treatment with HF compared to SB, while in the repaired ShB, the SBS was more enhanced by the surface treatment with SB compared to HF. The results of this study confirm the recommendation from the manufacturer, where SB was recommended for resin nanoceramics (ShB) and HF was recommended for PICN (VE). 

The results of the failure mode distribution revealed that VE and ShB without a surface treatment and whether immersed in DW or anti-COVID-19 mouthwash predominantly exhibited adhesive failure. The stress during the shear test concentrations at the interface of the aged RMCs and the adhesive, which had an insufficiently repaired SBS, resulted in predominant adhesive failure in the no surface treatment group. On the other hand, when the VE and ShB surfaces were treated with SB and HF, they showed predominantly cohesive failure in the composites. It is assumed that the surface treatment process used to enhance the adhesion, whether micromechanical or chemical, between the aged RMCs and the resin composites strengthened the adhesive bond, leading to failure susceptible to cohesive failure in the resin composite that has a lower elastic modulus compared to the RMCs.

Based on the results, although the anti-COVID-19 mouthwash used had an adverse adhesion between the repaired RMCs materials and the resin composites, the appropriate surface treatment protocol will achieve a clinically acceptable SBS. Therefore, clinicians should consider what kind of material is being repaired. The selection of the appropriate surface treatment protocols, as the manufacturer recommends, is beneficial to SBS. This research’s design, which concentrated on the use of a single universal adhesive, made it inapplicable to the use of other universal and traditional adhesives. Another limitation of this study was that it was not possible to stimulate the clinical conditions of repeated mouthwash use. Future studies should use more universal and traditional adhesives and long-term replicated oral environment conditions. In addition, the same mouthwash was used more than once when examining the durability and bond stability of the repaired RMCs. The bonding strength is just one factor that affects how well an adhesion technique works in clinics. Therefore, it is important to carefully analyze the results of our inquiry.

## 5. Conclusions

The adhesion throughout the RMCs repair process was impacted by the use of anti-COVID-19 mouthwashes. However, to achieve clinically acceptable SBS values, surface treatment procedures, as recommended by the manufacturers, were required for the RMCs prior to the repair with the resin composites.

## Figures and Tables

**Table 1:** Material compositions used in this experiment.

Material Name	Compositions	Lot Number
VITA Enamic (VITA Zahnfabrik, Bad Sackingen, Germany)	TEGDMA, UDMA Filler: Feldspar ceramic enriched with aluminum oxide, 86% by weight (75% by vol.) [24,25]	90001, 93140
Shofu Block HC (Shofu Inc., Kyoto, Japan)	TEGDMA, UDMA Filler: Zirconium silicate, Silica powder, micro fumed silica, 80% by weight (61% by vol.) [24,25]	0720695
Scotchbond Universal Plus adhesive; 3M ESPE, St. Paul, MN, USA	10-MDP, HEMA, vitrebond copolymer, filler, ethanol/water, initiators, silane, universal dual cure activator (separate vial), dimethacrylate resins containing BisGMA, APTES, and γ-MPTES	8904597
Filtek Z350 XT; 3M ESPE, St. Paul, MN, USA	TEGDMA, bis-GMA, bis-EMA, UDMA, silane treated silica, silane treated zirconia, sliane treated ceramic	NE81667
Povidone–iodine (PVP-I) (BETADINE^®^ Mouthwash/Gargle, Thai Meiji Pharmaceutical, Bangkok, Thailand)	Active ingredient: 7% W/V PVP-I Other ingredients: Glycerol, menthol, methyl salicylate, ethanol, saccharin sodium, and purified water	213033
Hydrogen peroxide (HP) (Siribuncha^®^ Siribuncha corporation, Bangkok, Thailand)	3% Hydrogen peroxide	02310146

Abbreviations: TEGDMA, triethylene glycol dimethacrylate; UDMA, urethane dimethacrylate; 10-MDP, 10-methacryloyloxydecyl dihydrogen phosphate; HEMA, 2-hydroxyethyl methacrylate; BisGMA, bisphenol A glycidyl dimethacrylate; APTES, (3-aminopropyl) triethoxysilane; γ-MPTES, 3-(triethoxysilyl)propyl ester.

**Table 2:** Three-way ANOVA results of the SBS.

Source	df	Mean Square	F	Sig.
material	1	2606.627	50.609	0.000
mouthwashes	2	374.390	7.269	0.001
surface treatment	2	5596.447	108.658	0.000
material * mouthwash	2	342.264	6.645	0.001
material * surface treatment	2	194.589	3.778	0.024
mouthwash * surface treatment	4	703.749	13.664	0.000
material * mouthwash * surface treatment	4	462.774	8.985	0.000

**Table 3:** SBS mean ± standard deviation (MPa) and percentage of failure mode.

Type of Ceramic	Mouthwashes	Surface Treatment	Mean SBS ± SD	Percentage of Failure Mode		
				Adhesive	Mixed	Cohesive
VITA ENAMIC	DW	No surface treatment	20.55 (6.52) ^ABC^	71.43	14.29	14.29
		HF	31.11 (8.94) ^DE^	0	14.29	85.71
		Sandblast	41.90 (8.17) ^F^	4.76	23.81	71.43
	PVP-I	No surface treatment	19.54 (5.49) ^AB^	100	0	0
		HF	40.93 (8.13) ^F^	14.29	4.76	80.95
		Sandblast	31.54 (7.49) ^DE^	14.29	9.52	76.19
	HP	No surface treatment	26.13 (8.20) ^BCD^	57.14	4.76	38.10
		HF	35.76 (7.00) ^EF^	0	19.05	80.95
		Sandblast	30.86 (5.80) ^DE^	0	0	100
Shofu Block HC	DW	No surface treatment	15.55 (6.92) ^A^	85.71	0	14.29
		HF	30.15 (9.45) ^DE^	33.33	0	66.67
		Sandblast	29.02 (7.27) ^DE^	9.52	9.52	80.96
	PVP-I	No surface treatment	13.62 (4.04) ^A^	100	0	0
		HF	25.59 (7.18) ^BCD^	4.76	23.81	71.43
		Sandblast	29.06 (7.12) ^DE^	4.76	14.29	80.95
	HP	No surface treatment	28.24 (7.53) ^CDE^	57.14	9.52	33.33
		HF	28.43 (5.14) ^DE^	19.05	23.81	57.14
		Sandblast	31.38 (6.65) ^DE^	4.76	33.33	61.90

The uppercase letter differences represent statistically significant differences at *p* < 0.05.

## Data Availability

Not applicable.
